# Recent advances in the diagnostic and therapeutic roles of microRNAs in colorectal cancer progression and metastasis

**DOI:** 10.3389/fonc.2022.911856

**Published:** 2022-10-13

**Authors:** Chen Liang, Jing-Bo Yang, Xin-Yi Lin, Bi-Lan Xie, Yun-Xian Xu, Shu Lin, Tian-Wen Xu

**Affiliations:** ^1^ Department of Digestive Tumours, The Second Affiliated Hospital of Fujian Medical University, Quanzhou, China; ^2^ Centre of Neurological and Metabolic Research, The Second Affiliated Hospital of Fujian Medical University, Quanzhou, China; ^3^ Group of Neuroendocrinology, Garvan Institute of Medical Research, Sydney, NSW, Australia

**Keywords:** colon cancer, microRNAs, progression, metastasis, therapeutic strategies

## Abstract

Colorectal cancer (CRC) is the third most common malignancy in the world and one of the leading causes of cancer death; its incidence is still increasing in most countries. The early diagnostic accuracy of CRC is low, and the metastasis rate is high, resulting in a low survival rate of advanced patients. MicroRNAs (miRNAs) are a small class of noncoding RNAs that can inhibit mRNA translation and trigger mRNA degradation, and can affect a variety of cellular and molecular targets. Numerous studies have shown that miRNAs are related to tumour progression, immune system activity, anticancer drug resistance, and the tumour microenvironment. Dysregulation of miRNAs occurs in a variety of malignancies, including CRC. In this review, we summarize the recent research progress of miRNAs, their roles in tumour progression and metastasis, and their clinical value as potential biomarkers or therapeutic targets for CRC. Furthermore, we combined the roles of miRNAs in tumorigenesis and development with the therapeutic strategies of CRC patients, which will provide new ideas for the diagnosis and treatment of CRC.

## Introduction

Colorectal cancer(CRC) is the third leading cause of cancer deaths in humans, with an overall incidence of approximately 5% and a 5-year survival rate of 40% to 60% (1). In 2018, there were approximately 1.8 million new CRC cases and 860,000 deaths. It is estimated that by 2040, the global CRC burden will increase by 72% to more than 3 million new cases, which will pose a serious threat to human health (2). Environmental and genetic factors play an important role in the pathogenesis of CRC (3). Dietary habits, smoking, low levels of physical activity, population ageing, and obesity are also factors that affect the pathogenesis of CRC (3). In recent years, although some progress has been made in the screening and treatment of CRC, the overall survival rate of patients with advanced stage disease remains low. Because the symptoms of CRC patients are not obvious in the early stage, and the prognosis is poor when it develops to the advanced stage, early detection and treatment are particularly important.

MicroRNAs are a group of single-stranded small noncoding RNAs of 21-23 nucleotides (nt) in length. They were first discovered and reported in 1993 (4), and an increasing number of studies have focused on the regulatory role of microRNAs since then. MicroRNAs play important roles in biological and pathological processes such as metabolism, apoptosis, differentiation, cell proliferation, cell cycle, invasion and metastasis, and are closely related to the occurrence and development of tumours. They regulate the expression of their target genes post transcriptionally and they may be involved in various physiological and pathological processes, including CRC metastasis, by affecting various factors in the human body (5).

Recent studies have shown that dysregulated microRNAs play an important role in the development and metastasis of CRC, and the abnormal expression of microRNAs may act as potential oncogenes or suppressors in the development of tumours. Disordered microRNAs may have carcinogenic or tumour suppressor functions, and can regulate some oncogenes and tumour suppressor genes. Similarly, they are also regulated by oncogenes and tumour suppressor genes (6). Studies have shown that alterations in the Wnt/β-catenin, EGFR, TGF β and TP53 signalling pathways can affect CRC survival, proliferation and metastasis, and specific miRNAs can lead to changes in these signalling pathways, thereby promoting or inhibiting tumorigenesis (7). The same microRNAs may act as a tumour promoter in one cancer and a tumour suppressor in another, so there is no need to study the role of the same microRNAs in different cancers. For example, miR-146a may have a carcinogenic effect in thyroid cancer and a tumour inhibitory effect in CRC (8).

As microRNAs could be used for the diagnosis and prognostic monitoring of CRC, their high tissue specificity and role in tumorigenesis make them novel biomarkers for diagnosing cancer and predicting patient outcomes (9). Meanwhile, due to the role of abnormal expression of microRNAs in tumour development and the therapeutic response, correcting miRNA deficiency or restoring miRNA function may be a new cancer treatment strategy.

In addition, the association of microRNAs with tumour angiogenesis, cell proliferation, metastasis, and apoptosis suggests that the related microRNAs may serve as potential targets for CRC therapy (10). This article reviews the roles of microRNAs in the occurrence, development and metastasis of CRC and provides new ideas for the diagnosis and treatment of CRC.

## Colorectal cancer

CRC is one of the most common gastrointestinal malignancies, the incidence of CRC in young adults is rapidly increasing (11). Patient survival is closely related to tumour stage at diagnosis, with approximately 50% of patients dying from distant metastases (12). The diagnosis of CRC is generally based on the evaluation of symptoms or screening. However, because CRC has no obvious symptoms in the early stage, most tumours have already metastasized at the time of diagnosis.

The treatment of CRC includes primary tumour resection, radiotherapy, chemotherapy, targeted therapy, immunotherapy and so on. Despite advances in surgery and adjuvant therapy, cure rates and long-term survival have barely changed over the past few decades (13). Decreased chemotherapy sensitivity remains a major obstacle preventing effective treatment of advanced disease. The development of cancer resistance to chemotherapy also often leads to treatment failure. Although there are targeted therapies for CRC, there are still relatively few ways to improve survival (14). Therefore, we need to clarify the mechanism of tumour progression and find new therapeutic targets. CRC patients are still at risk of recurrence after surgical removal of the tumour. Routine surveillance of postoperative patients to detect recurrence during the early asymptomatic period is one of the ways to improve survival (15).

## MicroRNAS

MicroRNAs are the most abundant small RNAs in animals and play a key role in the regulation of gene expression. They are involved in mRNA degradation by binding to the 3’-untranslated region (3’-UTR) and play important roles in cell differentiation, development, cell cycle regulation and apoptosis (6). It is estimated that microRNAs can regulate up to 30% of protein-coding genes in the human genome (16). Most microRNAs are detected in the cellular microenvironment, but circulating microRNAs or extracellular microRNAs can be detected in extracellular environments such as biological fluids. Circulating microRNAs exist as proteins or lipoprotein complexes in exosomes, microvesicles, apoptotic bodies, Argonaut protein complexes, and high-density lipoprotein complexes (17). These molecules are transported to recipient cells and regulate various physiological and pathological processes (18).

MicroRNAs are involved in the development and progression of cancer. Under specific conditions, microRNAs can act as both tumour promoters and tumour suppressors. Dysfunctional microRNAs can affect tumour progression, including maintaining proliferative signals, escaping growth inhibitors, resisting cell death, activating invasion and metastasis, and inducing angiogenesis (19). In recent years, an increasing number of studies have shown that microRNAs are not only potential biomarkers for CRC diagnosis and prognosis, but also potential therapeutic targets, and have broad application prospects in clinical diagnosis and treatment.

## The role of microRNAs in tumour progression

### Angiogenesis

Angiogenesis, the process of growing new blood vessels from venules of the existing capillaries, is an important step in tumour cell proliferation and metastasis (20). Studies have found that microRNAs can regulate all stages of angiogenesis (21). Approximately 33 different microRNA families have been reported to play a role in angiogenesis (22).

Zeng et al. (23) found that miR-25-3p secreted by CRC can be transferred to vascular endothelial cells through exosomes, destroy the integrity of the endothelial barrier, induce angiogenesis, and promote CRC metastasis. MTDH is a target gene of miR-375 in CRC. Han et al. (24) proved that the expression level of MTDH is negatively correlated with the expression of miR-375 in CRC. Inhibition of miR-375 expression in CRC can regulate cell proliferation and angiogenesis by increasing the expression of MTDH. Meanwhile, overexpression of miR-218 can significantly inhibit angiogenesis (25). In addition, miR-17~92 can inhibit CRC progression by inhibiting angio-genesis in tumours (26). Hu et al. (27) showed that exomiR-1229 has a positive effect on angiogenesis by activating the vascular endothelial growth factor (VEGF) pathway and may be a therapeutic target for inhibiting tumour angiogenesis. The recent findings of He et al. (28) revealed that miR-21-5p secreted by CRC cells is a key switch for cancer-induced angiogenesis and vascular permeability, and may also serve as a new target for cancer therapy. Moreover, hypoxia is closely related to angiogenesis. Targeting hypoxia-related microRNAs, such as miR-145, can inhibit CRC metastasis and may also help control tumour metastasis (29). In conclusion, the pathogenesis of cancer is related to the imbalance of angiogenesis, and miRNAs can regulate the related pathways of angiogenesis. Therefore, they are expected to become potential therapeutic targets for CRC.

### Premetastatic niche formation

The primary tumour creates a favourable microenvironment for subsequent metastasis in the secondary organs and tissues, that is, the premetastatic niche. The premetastatic niche can increase angiogenesis and vascular permeability, thereby promoting metastasis (30). Therefore, analysis of the molecular and cellular components of the premetastatic niche in blood may contribute to the diagnosis and prognosis of cancer metastasis. The study by Shao et al. (31) showed that during the development of CRC, miR-21 secreted by primary CRC cells is phagocytosed by macrophages in the liver, thereby forming a premetastatic niche in the liver, and circulating CRC cells can settle there and survive. A recent study demonstrated that upregulated miR-135a-5p plays a key role in CRC liver metastasis by promoting the formation of a premetastatic niche through dual regulation of immunosuppression and cell adhesion (32). Furthermore, circulating tumour-derived exosomal miR-203 can promote distant metastasis by inducing host M2 macrophages to form a premetastatic niche (33). Exosomal miR-25-3p is also involved in the formation of the premetastatic niche and may serve as a blood-derived biomarker for CRC metastasis (23). These studies show that miRNAs can participate in the formation of the premetastatic niche and promote CRC metastasis. Quantitative blood detection of the level of relevant miRNAs in circulating exosomes may be helpful for the diagnosis of CRC metastasis and the preventive treatment of high-risk metastatic patients.

### Cell proliferation and metastasis

Immortal proliferation of CRC cells is the basis of cancer development. MicroRNAs play an important role in the process of cell proliferation. Previous studies have shown that many microRNAs can affect the proliferation of CRC cells in different ways. For example, Huang et al. (34) found that upregulation of miR-17 could promote CRC proliferation. In contrast, miR-22 can inhibit the proliferation of CRC cells and slow the growth rate of tumours (35). In prostaglandin E2 (PGE2)-induced tumour cells, overexpression of miR-206 can reduce the proliferation of CRC cells (36), which may be a potential therapeutic target for PGE2-induced CRC cells. In addition, upregulation of miR-1258 and miR-500a-5p both inhibited tumour cell proliferation by blocking the cell cycle in G0/G1 (37, 38).

**Figure 1:**
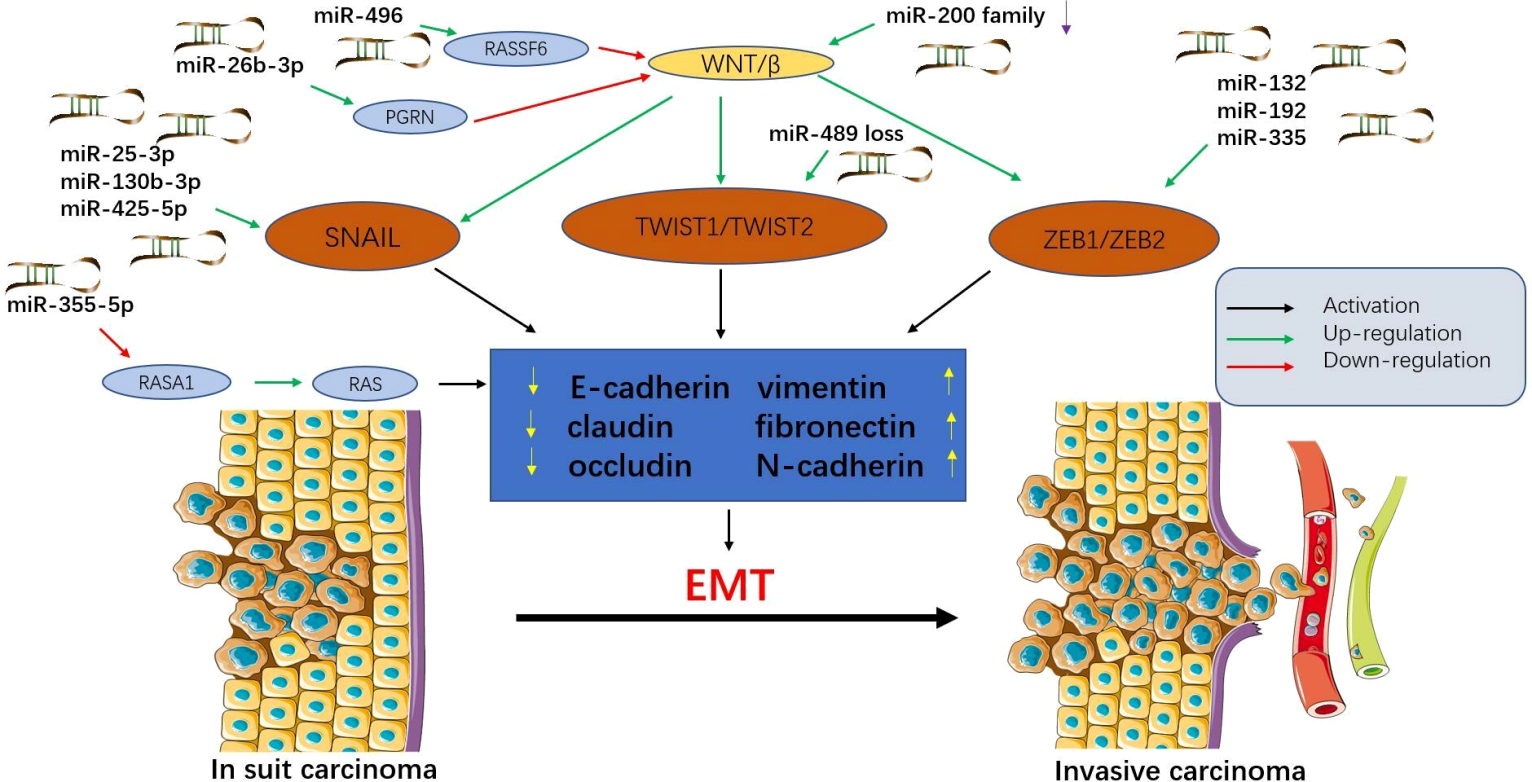
Epithelial-mesenchymal transition (EMT) is regulated by microRNAs in colorectal cancer (CRC). MicroRNAs affect multiple signalling pathways and participate in EMT by decreasing epithelial markers (e-cadherin, claudin, and occludin) and increasing interstitial markers (vimentin, fibronectin, and N-cadherin). During this process, epithelial cells acquire mesenchymal phenotypes, which play an important role in the progression and metastasis of CRC. Tumour cells that have undergone EMT can invade the local stroma and enter the vasculature, travel in the circulation, and finally establish a secondary tumour at a distant site. This figure summarizes some microRNAs involved in the EMT process in CRC.

MicroRNAs can control multiple aspects of epithelial-mesenchymal transition (EMT) and mesenchymal-epithelial transition (MET) and support tumour progression and metastasis (39) (**Figure 1**). Exosomes from tumour cells can transfer miRNAs to normal cells, stimulating carcinogenesis and promoting metastasis (40). Exosomes promote EMT by targeting RASp21 protein activator 1 (RASA1) to deliver miR-NA-335-5p, thereby promoting CRC cell invasion and metastasis (41). In addition, miR-29b-3p can directly target progranulin (PGRN) to alter the downstream Wnt signalling pathway and promote EMT (42). The miR-496/RASSF6 axis can also promote EMT and CRC migration through Wnt signalling (43). The study by Wang et al. (44) showed that miR-25-3p, miR-130b-3p, and miR-425-5p can induce tumour cell proliferation and metastasis, and may be potential therapeutic targets for blocking CRC metastasis.

In addition to cancer-promoting microRNAs, there are also cancer-suppressing microRNAs. Upregulation of miR-200c can inhibit EMT, thereby inhibiting tumour progression (45). Furthermore, the expression of miR-382-5p was significantly down-regulated in CRC tissues and cell lines. Upregulation of miR-382-5p expression can target NR2F2 and PD-L1, thereby inhibiting CRC cell proliferation and metastasis (46, 47). Przygodzka et al. (48) reported that miR-192 and miR-194 can inhibit snail-induced EMT and metastasis. Therefore, prevention of EMT may be a promising approach to block CRC metastasis. In a word, under normal physiological conditions, miRNAs can maintain the normal regulation of some cellular processes, and their abnormality will lead to abnormal growth and biosynthesis of cells, thus promoting or inhibiting the spread and metastasis of tumors.

### Apoptosis

Apoptosis is a programmed death process that occurs during normal cell development and senescence. Chemotherapy forces cancer cells to undergo apoptosis by causing DNA damage or cell damage. Abnormal apoptosis is one of the pathogenic mechanisms of CRC and plays a role in the resistance to chemotherapeutic drugs and radiotherapy (49). MicroRNAs play an important role in tumour cell apoptosis and drug resistance. Activation of the caspase family of proteases is the main pathway for inducing apoptosis (50). MiR-433 can increase the expression of caspase-3 and caspase-9, thereby promoting apoptosis (51). Overexpression of miR-218 can also promote CRC cell apoptosis by increasing caspase-8 levels (52). In the Kyoto Encyclopedia of Genes and Genomes (KEGG) apoptosis pathway, miR-92a is associated with two apoptosis-related genes, CSF2RB and BCL2L1. Moreover, increased expression of miR-92a-3p in tumour tissue can improve patient survival time (53). Overexpression of miR-766 reduces CRC cell growth and induces apoptosis by inhibiting the MDM4/p53 pathway (54). MiR-27a-3p increases apoptosis through the ERK-MAPK pathway, while miR-422a induces apoptosis in CRC cells through the p38-MAPK pathway (55, 56). In contrast, mi-421 exerts an anti-apoptotic effect in CRC by downregulating caspase-3 (57). Therefore, the regulation of microRNAs will help to regulate the occurrence and development of CRC, promote cancer cell apoptosis, and alleviate drug resistance.

### Immune system activity

Escape from immune system surveillance is an important link in tumorigenesis and development. Studies have shown that microRNAs may be involved in the immune escape process of CRC and are significantly associated with tumour survival. MicroRNAs may be involved in the differentiation of monocytes into M2 macrophages, which have been implicated in playing key roles in colon cancer (58, 59). Exosomes derived from M2 macrophages transfer miR-21-5p and miR-155-5p to CRC cells, promoting cell migration and invasion (60). The results of Ma et al. showed that M2 macrophage-derived exosomal miR-155-5p could promote immune escape by colon cancer, enhancing the progression of CRC (61). Studies have shown that miR-203-containing exosomes released by CRC cells can be internalized by monocytes, thereby promoting the expression of M2 markers (33). However, the PD-1/PD-L1 pathway, as an important immune checkpoint, is dysregulated in various human malignancies, including CRC, and is involved in tumorigenesis by inhibiting antitumor immune response. MiR-124 inhibits PD-L1 expression in CRC cells, which in turn promotes T-cell mediated anti-cancer responses (62). In conclusion, the interaction between miRNAs and immune checkpoints has great application prospects in the personalized treatment of CRC in the future.

### Impact on the tumour microenvironment

Tumour growth and metastasis are highly dependent on the interaction between tumour and relevant microenvironment, and several miRNAs have been shown to play a key role in the interaction between tumour and tumour microenvironment (TME). In every step of tumour growth and metastasis, complex molecular interactions occur between cells in the tumour microenvironment, such as fibroblasts and immune-related cells (63). Cancer-associated fibroblasts (CAFs) affect tumour growth by regulating inflammation or direct cell-to-cell communication. Studies have shown that miRNA can alter chemokines secreted by fibroblasts to alter TME, thereby promoting migration and invasion (64). Tumour-derived microRNAs affect the matrix and immune cell components of the tumour microenvironment. In TME, miRNA is considered to be an important molecular mechanism for the interaction between tumour cells and immune cells. For example, miRNAs can control the production of chemokines or cytokines by tumour cells, which in turn affect the aggregation and expansion of immune cells (65). Tumour-associated macrophages (TAMs) are the key components of TME, and miRNAs play an important role in the regulation of TAMs on tumour progression. TAMs have been shown to be associated with a poor prognosis of CRC. TAMs can induce EMT in CRC cells by regulating the STAT3/miR-506-3p/FoxQ1 axis, thereby promoting metastasis (66). However, miR-195-5p could inhibit the polarization of M2-like TAMs, and patients with low miR-195-5p levels have significantly shorter overall survival times (67).

Mesenchymal stem cells (MSCs) are also an important part of the TME and play a key role in promoting tumour progression (68). In the TME, microRNAs generally have tumour-promoting effects and are an important direction for future cancer therapy. Although MSCs have some antitumor activity, microRNAs mediate immunosuppressive activity (69), which provides ideas for future cancer therapy. Intestinal microRNAs can influence the growth and composition of the intestinal microbiota (70). The pathogenesis of CRC is also associated with disorder in the microbiota, termed ecological disorder (71). Imbalances in microRNAs can affect the survival or gene expression of some beneficial bacteria in the microbiota. Dysfunctional microRNAs in tumour cells can be transmitted to stromal cells and immune cells, creating a more favourable microenvironment for tumour cells (72). Thus, microRNAs can modulate the microbiota, promoting the growth of beneficial bacteria and inhibiting the growth of cancer-causing bacteria. In short, the interaction between microRNAs and the TME may also be one of the entry points for antimetastatic treatment in the future.

## The role of microRNAs in tumour tumourigenesis

MiRNAs also play an important role in the initiation of human cancer. MiRNAs are related to the pathogenesis of various types of human malignant tumours. In several types of cancer, the decreased expression of miR-34 and let-7 can trigger tumorigenesis, and the up-regulation of miR-34 and let-7 can lead to tumour growth inhibition (73). Moreover, there is ample evidence that miRNAs are closely related to the dysregulation of several key pathways in CRC. miR-31 is a potential driver of colon tumorigenesis by targeting EphB2 and EphA2 signalling pathways (74). Mamoori et al. (75) demonstrated that miR-21 expression was increased many times in colonic cancer stem cells compared to parental cells. Moreover, since the expression of miR-21 is increased, the expression level of PTEN in the colon bulb is decreased, and the Akt signalling pathway is activated, miR-21 is considered to play an important role in the tumorigenic regulation of colon cancer stem cells.

Inflammation also drives the steps of tumorigenesis. Jeffries et al. (76) found that miR-223 can regulate tumorigenesis at multiple levels, including by inhibiting the inflammatory tumour microenvironment and regulating the malignancy of cancer cells. And some studies have proved that the level of miR-223 can be used to predict the probability of CRC by sequencing circulating exosomal miRNAs (77). MiRNAs can be used as therapeutic targets and prediction means, and are potential tools for cancer management and treatment in the future. However, more research is needed before they can be applied to clinic.

## Clinical applications

### Early diagnosis

**Figure 2:**
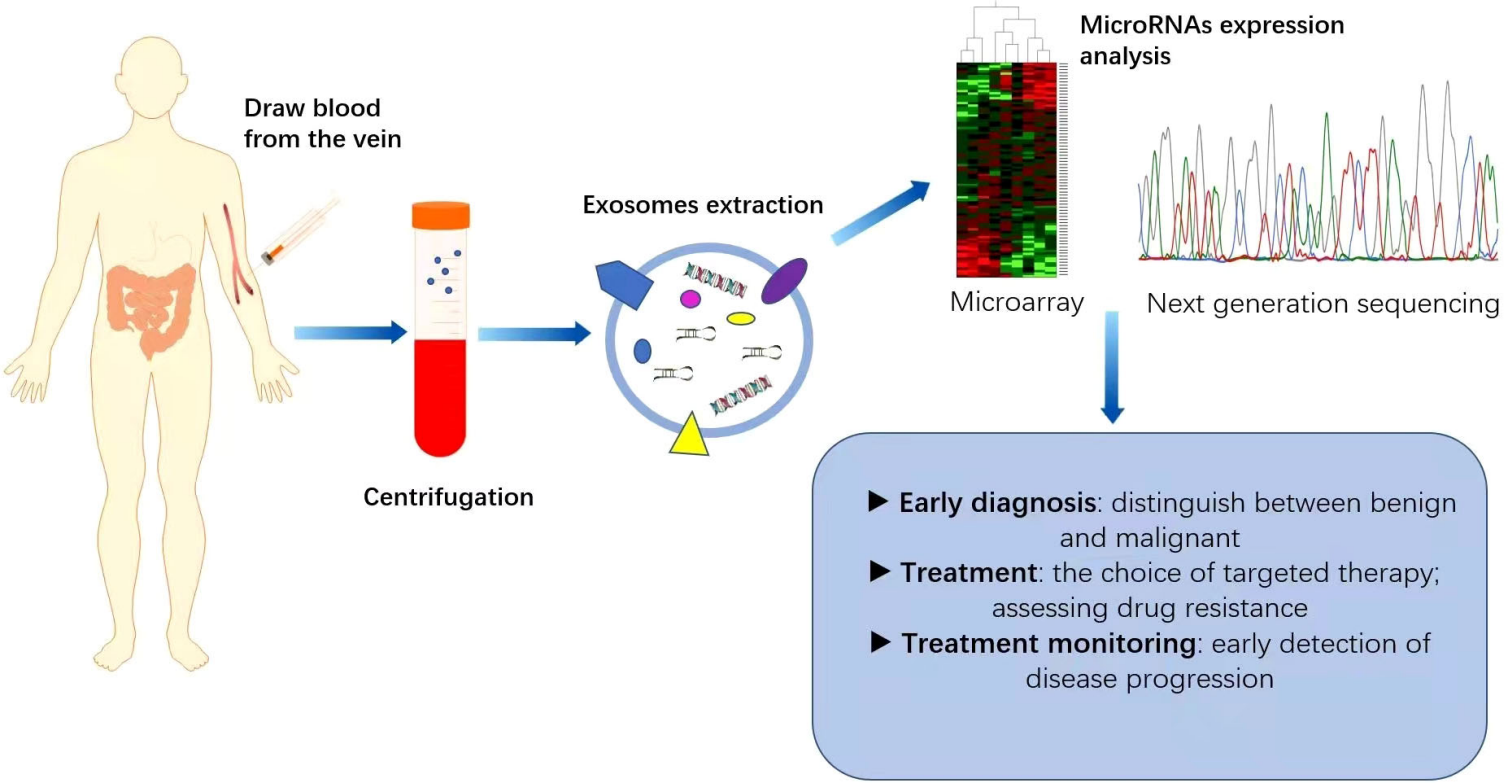
Application of miRNAs as biomarkers for colorectal cancer (CRC). MicroRNAs are generally expressed abnormally in CRC patients. Blood samples collected from CRC patients are used as a source of circulating exosomes and after their isolation, we can analyse the pattern of microRNA expression, which is helpful for the diagnosis and treatment of CRC. This can play an important role in the early diagnosis of cancer, identification of high-risk patients requiring intensive treatment, monitoring of drug efficacy and real-time monitoring of the effectiveness of treatment.

Increasing evidence suggests that miRNAs can serve as non-invasive biomarkers for CRC diagnosis and prognosis (**Figure 2**). They exist in the bloodstream in a highly stable form by binding to specific proteins or vesicles (78, 79). Karimi et al. (80) showed that miR-23a and miR-301 were upregulated in patients compared with healthy individuals, which can be used to distinguish CRC patients from normal subjects. Zhu et al. (81) found that miR-19a-3p, miR-21-5p and miR-425-5p were significantly upregulated in CRC patients compared with healthy individuals. Cheng et al. (82) found that the circulating abundance of exocrine miR-146a correlated with high levels of CD66 neutrophils. However, the proportion of tumour-infiltrating TCD8 cells decreased. MiR-146a is the main miRNA in the exosomes of CRC stem cells and can be used as a diagnostic biomarker. In addition, both miR-486-5p and miR-18b-5p have potential for use as non-invasive biomarkers for the early diagnosis of CRC (83, 84). Min et al. (85) found that miR-92b was differentially expressed in CRC patients and healthy individuals but could not be used to differentiate between CRC and adenoma. Even so, it has promise as a minimally invasive tool for the early diagnosis of CRC. In addition, miR-21, miR-155, and miR-221, which are expressed differently in colon and rectal cancers, can be used to distinguish colon and rectal cancer (86). In addition, the levels of miR-17-5p and miR-92a-3p isolated from serum exosomes were found to correlate with the pathological stage and grade of patients with CRC (87).

Numerous studies have shown that microRNAs in serum, exocrine and even faeces have the potential for early diagnosis. Decreased expression of miR-4478 and miR-1295-p in stool specimens is a noninvasive and effective diagnostic marker for CRC patients, which can be detected at an early stage of CRC, suggesting that it may be a promising CRC screening approach (88). Moody et al. (89) found that miR-20a in the faeces of CRC patients also serves as a potential prognostic biomarker. Furthermore, stool miR-135b-5p is not only a potential biomarker but also an ideal candidate intervention strategy for CRC patients (90). The establishment of appropriate miRNA biomarkers is very important for the early diagnosis of CRC. Of course, prospective studies with larger patient cohorts are needed to confirm the diagnostic value of these microRNAs. Further efforts are required before microRNAs in faeces can be used clinically.

Treatment options for patients with CRC require accurate assessment of TNM staging. Therefore, biomarkers that can accurately predict preoperative TNM staging will significantly improve the treatment efficiency of CRC. Bjørnetrø et al. (91) found that low levels of miR-486-5p and miR-181a-5p were associated with locally advanced dis-ease and lymph node metastasis, while high levels of miR-30d-5p were associated with metastatic progression. Orosz et al. (86) also evaluated the potential of several microRNAs to distinguish individual TNM stages. The results showed that the expression levels of miR-155, miR-34a, and miR-29a in the serum of TNMII, III, and IV patients were downregulated.

### Treatment of CRC

#### Drug resistance

Although good progress has been made in the systemic treatment of tumours in recent years, in addition to surgery, chemotherapy is still the main treatment for CRC. The resistance of cancer cells to chemotherapy is a major factor leading to chemotherapy failure, often resulting in a poor prognosis. Many studies have shown that there is a certain relationship between tumour drug resistance and microRNA imbalance. Tumour drug resistance can occur through a variety of mechanisms, including apoptosis inhibition (92). Studies have shown that ectopic expression of miR-520 g resists 5-FU-induced apoptosis by inhibiting the expression of p21 (6). Decreased levels of miR-125b-5p have also been shown to contribute to tumour cell metastasis and 5-FU chemotherapy resistance (93). Similarly, miR-22 and miR-206 can also promote apoptosis induced by 5-FU (94, 95). Recent studies have shown that the tumour suppressor miR-27b-3p can increase the sensitivity of CRC cells to 5-FU (96). Oxaliplatin (OXA) resistance is also a major obstacle to the treatment of advanced CRC. Li et al. (97) reported that miR-34a was significantly downregulated in OXA-resistant patients, which could reduce OXA resistance by targeting OAZ2. In addition, studies have shown that miR-128-3p can enhance tumour sensitivity to chemotherapy and may become a promising OXA chemotherapy marker (98). In contrast, mir-5000-3p, mir-135b-5p, and mir-208b were associated with decreased sensitivity to OXA chemotherapy (99–101). Recent studies have shown that miR-24-3p can enhance the resistance of CRC cells to methotrexate (MTX) (102).

The hypothesis that drug resistance is the result of tumour-host interactions has been proposed, suggesting new strategies for overcoming the development of cancer chemotherapy resistance (103). Studies have shown that miR-21 and 5-FU combined with engineered exosomes can effectively reverse the drug resistance of 5-FU-resistant colon cancer cells and improve therapeutic efficiency (104). More efforts are needed to prevent cancer cells from developing resistance to chemotherapy and to try to resensitize cancer cells to chemotherapy drugs (**Table 1**).

**Table 1:** Several microRNAs are involved in CRC resistance.

microRNA	Effect on drug resistance	Type of drug	Target(s)	Reference
miR-520g	Inhibit	5-FU	P21	(6)
miR-125b-5p	Inhibit	5-FU	Sp1, CD248	(93)
miR-22	Inhibit	5-FU	HDACs	(94)
miR-206	Inhibit	5-FU	Bcl-2	(95)
miR-34a	Inhibit	OXA	OAZ2	(97)
miR-128-3p	Inhibit	OXA	Bmi1,MRP5	(98)
miR-5000-3p	Promote	OXA	USP49	(99)
miR-135b-5p	Promote	OXA	MUL1	(100)
miR-208b	Promote	OXA	PDCD4	(101)
miR-24-3p	Promote	MTX	CDX2	(102)

There are two different types of CRC: “microsatellite stability” (MSS) and “microsatellite instability” (MSI). Cancers of MSS and MSI types promote tumorigenesis and progression through two distinct molecular pathways (105). Microsatellite stability-high (MSI-H) is caused by functional defects in the DNA mismatch repair (MMR) system. MSI-HCRC immune checkpoint molecules, such as PD-1 and PD-L1, have been shown to be resistant to the antitumor immune response (106). MicroRNAs can play a role in cancer-related immune responses by targeting immunosuppressive or immunostimulatory factors. It has been proven that miR-140-3p, miR-382-3p, miR-148a-3p, miR-93-5p, miR-200a-3p, miR-200c-3p, miR-138-5p and miR-15b-5p can regulate immune escape by inhibiting tumour PD-L1. They can also transform the immunosuppressive tumour microenvironment into a proinflammatory tumour microenvironment, enhancing the chemosensitivity of tumour cells (107). Therefore, it may be possible to alleviate the drug resistance of MSI-H CRC by regulating microRNAs.

Long noncoding RNAs (lncRNAs) are noncoding RNAs (ncRNAs) and microRNAs. Studies have shown that lncRNAs, as precursors of microRNAs, are also associated with drug resistance. For example, the lncRNA MIR100HG, a precursor of miR-100 and miR-125b, can lead to cetuximab resistance (108). The lncRNA-XIST/miR-125b-2-3p axis can also induce chemoresistance in CRC, but the specific mechanism by which it affects chemosensitivity has not been elucidated (109). The complex feedback loop between lncRNAs and microRNAs may provide new perspectives for the reversal of CRC drug resistance. In contrast, the lncRNA-XIST/miR-137 axis can enhance CRC glycolysis and chemotherapy resistance, providing a possible alternative to improve chemotherapy efficacy in CRC patients (110).

#### Therapeutic target and microRNA therapy

The aberrant expression of microRNAs plays an important role in the development of cancer and the response to anticancer drugs. Correcting microRNA defects or restoring microRNA function can be used as a new cancer treatment strategy. MicroRNAs have been proven to be therapeutic targets for CRC (111). For example, miR-135b has been shown to be upregulated in CRC and associated with tumour progression and a poor clinical prognosis. Therefore, tumour growth can be inhibited by reducing miR-135b. Studies have shown that blocking exocrine miR-25-3p in CRC can reduce the vascular permeability and metastasis of CRC, suggesting that miR-25-3p can be used as a therapeutic target for interfering with CRC metastasis (23).

With the development of high-throughput sequencing technology, the interaction of the gene expression network system comprised of messenger RNAs (mRNAs), miRNAs, lncRNAs and circular RNAs (circRNAs) in CRC progression has been discovered. It has been proven that lncRNA-miRNA cross-talk is a novel mechanism affecting CRC cell proliferation, invasion and metastasis (112). For example, lncRNA TUG1 can promote the growth and migration of CRC cells by secreting miR-145-5p, and the TUG1/miR-145-5p/TRPC6 pathway can serve as a target for CRC diagnosis and therapy (113). Liu et al. showed that the circIFT80/hsa-miR-370-3p/WNT7B signalling axis might also play a role in carcinogenesis (114). CircIFT80 inhibits the expression of hsa-miR-370-3p in CRC cell lines, thereby inhibiting apoptosis. Therefore, in addition to research on microRNAs, research on lncRNAs and circRNAs may also provide new ideas for the targeted therapy of CRC.

In recent years, the application of immune checkpoint inhibitors (ICIs), especially anti-PD-1 therapy, has greatly improved the efficiency of tumour treatment. However, the role of ICIs in CRC is generally limited to MSI-H tumours. The latest study by Liu et al. (115) found that miR-15b-5p downregulated the expression of PD-L1 at the protein level, inhibited tumorigenesis, and improved the sensitivity to anti-PD-1 therapy. Elevating the level of miR-15b-5p can improve the sensitivity of MSS CRC patients to ICI treatment. Blocking oncogenic microRNAs may adversely affect the physiological functions regulated by these microRNAs, thus requiring specific sites or cellular targets to avoid potential adverse effects. At the same time, extensive clinical trials are needed to evaluate the efficacy and safety of microRNAs as therapeutic targets in patients.

Despite advances in the application of immune checkpoint blockade therapy in malignancies, CRC patients usually only benefit if they have tumours with mismatch repair deletions or severe mutations in MSI-H (116). However, most tumours are MSS, so immunotherapy has a low response rate in treating CRC. Many studies have shown that microRNAs can modulate immune responses, and some of these microRNAs can inhibit the progression of CRC and are expected to be effective antineoplastic drugs. Since the disorder of microRNAs was first discovered in cancer, it has been studied extensively and uncovered new therapeutic possibilities. MiRNAs can regulate multiple signalling pathways of the immune system and have the advantage of multiple targets (117). Previous studies have shown that restoration of miR-34 expression can reduce the proliferative potential of CRC cells; thus, miR-34 can be used as a therapeutic drug (118). In addition, miR-34 can also increase tumour sensitivity to 5-Fu, thereby reversing drug resistance (119). Unfortunately, the therapeutic application of microRNAs is limited by technical barriers. MicroRNA molecules are unstable and are rapidly cleared from the blood, with only a small fraction absorbed by cells (120).

In some studies, exosomes have been used as transporters for microRNA drugs, and the lipid bilayer membrane of exosomes can protect exosomes from being degraded during blood circulation. Han et al. (121) used CBMSC-derived exosomes to infiltrate anti-miRNA-221 into solid tumours and significantly inhibited tumour growth. As a tumour suppressor microRNA, miR-124 can regulate several oncogenes and signalling pathways closely related to tumour growth and promote T-cell dependent immune responses. The study by Rezaei et al. (1) used CT-26-derived exosomes as a natural vehicle for miR-124-3p delivery, which elicited potent antitumor immune responses and reduced tumour growth. In the future, the response rate of immunotherapy may be significantly improved by increasing the technology of exosomes carrying microRNAs. However, the source of exosomes is limited and lacks targeting, there are still many challenges in future applications, and further research is needed. In addition, the efficacy and safety of microRNA therapy in patients need to be studied.

### Prognosis of colon cancer

#### Predicting recurrence

Approximately one-third of patients with CRC undergoing radical surgery will experience disease recurrence (13). Studies have shown that miRNAs can be used as biomarkers for predicting CRC recurrence, which is beneficial to the prognosis of CRC patients. The serum levels of exocrine miR-1229, miR-1224-5p, miR-223, let-7a, miR-150 and miR-21 in CRC patients were significantly increased, and then they decreased after resection (122). Plasma miR21-5p could be used to predict recurrence and disease progression after surgical resection (123). Studies have shown that serum exocrine miR-21 could be used to predict CRC recurrence and a poor outcome in TNM stage II, III, or IV (124). In addition, postoperative plasma miR-31, miR-141, and miR-16 have also been shown to be biomarkers of disease recurrence after surgical resection (125). In general, for patients with stage II CRC, surgical resection of the primary tumour is effective and may not require other treatment, but whether adjuvant chemotherapy should be used in patients with stage II CRC remains controversial (111). Yamazaki et al. (126) proposed that high expression of miR-181c plays a role in predicting recurrence of stage II CRC. Through the study of microRNAs, it is possible to assess which postoperative patients with stage II CRC may benefit from adjuvant therapy (**Table 2**).

**Table 2:** Examples of microRNAs associated with CRC prognosis.

microRNA	Clinical application	reference
miR21-5p	Predicting recurrence after surgical resection as well as disease progression	(123)
miR-21	Prediction of CRC recurrence and poor prognosis when stratified by TNM stage II, III or IV; Prediction of clinical response and outcome in patients treated with chemoradiotherapy	(124, 127)
miR-31	Biomarkers of disease recurrence after surgical resection	(125)
miR-141	Biomarkers of disease recurrence after surgical resection	(125)
miR-16	Biomarkers of disease recurrence after surgical resection	(125)
miR-181c	Prediction of CRC recurrence with TNM stage II	(126)
miR-194	Potential predictive biomarkers of chemotherapy response	(128)
miR-33a-5p	Predictive markers of chemotherapy efficacy	(129)
miR-99b	Prediction of clinical response and outcome in patients treated with chemoradiotherapy	(127)
miR-375	Prediction of clinical response and outcome in patients treated with chemoradiotherapy	(127)

Aberrant expression of microRNAs as biomarkers may contribute to individualized treatment of patients. A study by D’Angelo et al. (128) showed that miR-194 was a potential predictive biomarker of chemotherapy response. Meanwhile, other studies have found that miR-33a-5p, miR-21, miR-99b, and miR-375 can predict clinical response and outcomes in patients treated with radiotherapy and chemotherapy (127, 129). Yin et al. (130) established an *in vitro* tumour model called patient-derived tumour-like cell clusters (PTCs), which has been shown to be useful for assessing tumour sensitivity to drugs. By incorporating microRNAs as markers into this predictive model, real-time efficacy monitoring can be achieved to assess the benefit of chemotherapy or targeted therapy.

#### Metastasis of colon cancer

**Figure 3:**
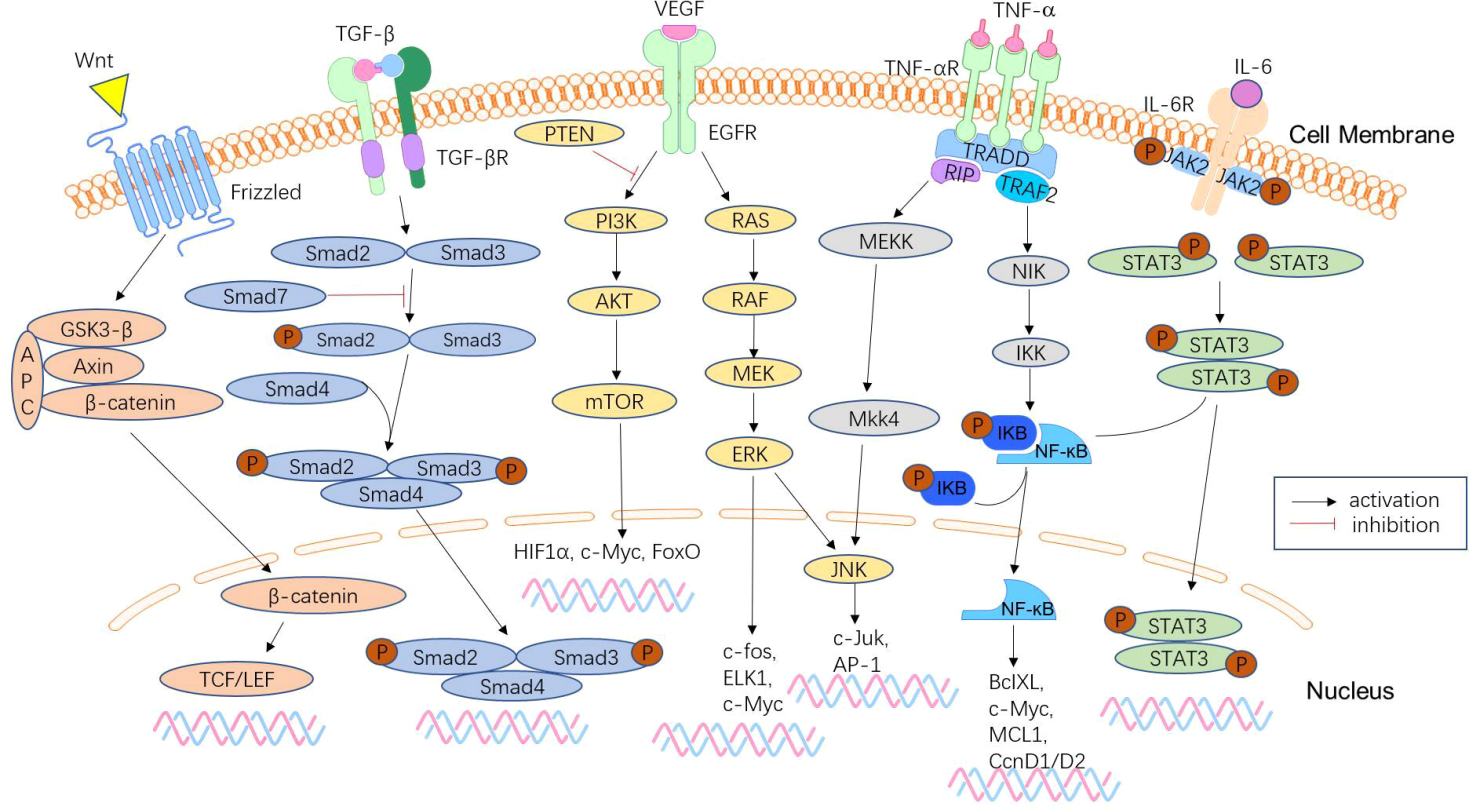
Signalling pathways involved in CRC metastasis. CRC metastasis is mediated by a complex network of signalling pathways, which include the Wnt/β-catenin signalling pathway, TGF-β/Smad pathway, phosphoinositide 3-kinase (PI3K)/phosphatase and tensin homologue (PTEN)/AKT pathway, KRAS-ERK signalling pathway, NF-κB signalling pathway, and JAK/STAT3 signalling pathway. These pathways lead to tumour anti-apoptosis, EMT, proliferation, and invasion.

Approximately 50% of CRCs will metastasize in the advanced stage of malignant tumours, and distant metastasis is the main cause of death of CRC patients (**Figure 3**). Early detection and treatment of distant metastasis is of great significance to improve the long-term survival of CRC patients. MicroRNAs are significantly associated with tumour metastasis. Several microRNAs, including members of the miR-34 and miR-200 families, have been found to target the mRNAs of EMT transcription factors, such as ZEB1, ZEB2, and SNAIL (131). Downregulation of these microRNAs is associated with distant metastasis and advanced tumours. The liver is the most common metastatic site of CRC. The study by Hur et al. (132) showed that elevated serum miR-203 levels are closely associated with liver and systemic metastasis. Teng et al. (133) detected significantly elevated plasma miR-193a levels in CRC patients with liver metastasis. Lan et al. (60) found that miR-21-5p and miR-155-5p were transferred to CRC cells *via* exosomes and were key factors in promoting CRC metastasis. Preventing such messages may be a new strategy to suppress CRC metastasis. These microRNAs can be used as biomarkers to determine prognosis and predict distant metastasis. MiR-181a is significantly upregulated in CRC tissues of patients with liver metastases and promotes tumour cell growth and proliferation, which is closely associated with distant metastasis and poor survival (134). In contrast, miR-802 is negatively correlated with lymphatic and distant metastasis of CRC (135), and may be a regulatory target for suppressing metastasis.

#### Induction of muscular dystrophy

Cachexia is a complex metabolic and behavioural syndrome associated with underlying disease and is characterized by loss of skeletal muscle. Previous studies have found a significant correlation between skeletal muscle mass and circulating miR-21 expression in CRC patients, suggesting that assessment of serum miR-21 levels can be used to assess the risk of sarcopenia and cancer cachexia in patients with CRC (136). The results of Miao et al. (137) suggest that abundant microRNAs in tumour exosomes may induce muscle atrophy mainly by targeting Bcl-2-mediated apoptosis. In addition, the detection of serum miR-203 expression can be used to evaluate the risk of sarcopenia, and miR-203 may be a new therapeutic target for inhibiting sarcopenia in patients with CRC (138).

## Conclusion and perspectives

MicroRNAs have a wide range of biological functions and are involved in many physiological and pathological processes, including cancer. An increasing number of studies have shown that microRNAs play an important role in the progression and metastasis of CRC. Specific microRNAs can be used to overcome diagnostic and therapeutic challenges of different types of tumours. The combination of novel microRNA markers with traditional biomarkers may help to improve the specificity and sensitivity of detection. Using microRNAs as new therapeutic targets to correct maladjusted microRNAs would be a promising approach for CRC therapy. In future studies, we should determine which biological fluids and assays are most suitable for CRC screening and which microRNA combinations have the best diagnostic performance. We should maximize the specificity of these microRNA biomarkers. At the same time, we should increase our understanding of the role of microRNAs in the molecular pathogenesis and treatment of cancer. This will facilitate the clinical application of microRNAs.

At present, some progress has been made in the study of microRNAs reversing drug resistance, but there are still few studies on immunotherapy resistance in MSI-H CRC. In addition, the biggest problem facing microRNA therapy is the choice of carrier. Nanoparticles or exosomes are used as carriers in the current studies. Both of these carriers have certain limitations, and more research is needed to overcome these difficulties and allow for their application in clinical practice. The roles and functions of individual microRNAs in CRC remain unclear and more research is needed. Investigating the effects of microRNAs on the occurrence, development and metastasis of CRC is of great significance for the diagnosis and treatment of CRC.

lncRNAs, circRNAs and microRNAs are all ncRNAs and have great potential in clinical applications. Accumulating evidence suggests that a complex regulatory net-work exists between lncRNAs, circRNAs, and microRNAs. They have great biological potential and may regulate CRC initiation, progression and metastasis. However, the exact mechanisms of how these interactions affect tumorigenesis and progression have not been fully revealed. Future analysis of different RNA molecules with potential crosstalk may provide new insights into the diagnosis and treatment of CRC, contributing to the improvement of biomarker prediction and the development of new treatments.

## Author contributions

All authors contributed to the article and approved the submitted version.

## Funding

We are supported by the National Natural Science Foundation of China (grant number 81970791), Science and Technology Bureau of Quanzhou (grant number 2020CT003), Fujian Province Scientific Foundation (grant number 2019J01168), and the Young and middle-aged backbone talent foundation of Fujian Provincial Commission of Health Construction (grant number 2020GGA058).

## Conflict of interest

The authors declare that the research was conducted in the absence of any commercial or financial relationships that could be construed as a potential conflict of interest.

## Publisher’s note

All claims expressed in this article are solely those of the authors and do not necessarily represent those of their affiliated organizations, or those of the publisher, the editors and the reviewers. Any product that may be evaluated in this article, or claim that may be made by its manufacturer, is not guaranteed or endorsed by the publisher.

## Abbreviations

CRC, colorectal cancer; 3’-UTR, 3’-untranslated regions; VEGF, vascular endothelial growth factor; PGE2, prostaglandin E2; EMT, epithelial-mesenchymal transition; MET, mesenchymal-to-epithelial transition; RASA1RASp21, protein activator 1; PGRN, progranulin; PD-1, programmed death-1; KEGGKyoto, Encyclopedia of Genes and Genomes; PD-L1, Programmed death ligand 1; MSCs, mesenchymal stem cells; TME, tumor microenvironment; TAMs, tumor-associated macrophages; 5-Fu, 5-Fluorouracil; OXA, Oxaliplatin; OAZ2O, rnithine Decarboxylase Antizyme 2; MTX, methotrexate; MSS, microsatellite stability; MSI, microsatellite instability; MSI-H, microsatellite instability-high; MMR, mismatch repair; lncRNAs, long non-coding RNAs; ncRNAs, non-coding RNAs; ICI, simmune checkpoint inhibitors; mRNAs, messenger RNAs; circRNAs, circleRNAs; PTCs, patient-derived tumor-like cell clusters.
